# Evaluation of T Cell Immunity against Human Cytomegalovirus: Impact on Patient Management and Risk Assessment of Vertical Transmission

**DOI:** 10.1155/2016/9384813

**Published:** 2016-12-01

**Authors:** Giulia Freer, Paola Quaranta, Mauro Pistello

**Affiliations:** Retrovirus Center, Department of Translational Research, University of Pisa, Via del Brennero 2, 56127 Pisa, Italy

## Abstract

Cytomegalovirus (CMV) is one of the most common infectious agents, infecting the general population at an early age without causing morbidity most of the time. However, on particular occasions, it may represent a serious risk, as active infection is associated with rejection and disease after solid organ transplantation or fetal transmission during pregnancy. Several methods for CMV diagnosis are available on the market, but because infection is so common, careful selection is needed to discriminate primary infection from reactivation. This review focuses on methods based on CMV-specific T cell reactivity to help monitor the consequences of CMV infection/reactivation in specific categories of patients. This review makes an attempt at discussing the pros and cons of the methods available.

## 1. Introduction

Cytomegalovirus (CMV) infects roughly 50% of the population in industrialized countries by adulthood. In developing countries, infection rate is much higher, leaving few adults seronegative [1]. Most new infections do not reach clinical relevance, but in specific instances CMV may represent a risk. For instance, rejection after transplantation may be associated with CMV infection or reactivation. In addition, transmission of CMV to fetus during pregnancy is one of the most frequent causes of deafness, to name but one of the consequences possible [2]. For different reasons, in both of these situations, it is quite relevant to assess whether CMV viremia is due to new infection or reactivation of latent CMV. Several methods for CMV diagnosis are available on the market, spanning almost the whole range of possible formats [3]. Certain methods may be useful to discriminate between primary infection or reactivation. To help assess the real risk of severe disease posed by CMV, determination of T cell reactivity is being evaluated by several clinical researchers.

## 2. CMV Pathology

CMV belongs to Herpesviridae and is the prototype of the Betaherpesvirinae subfamily group. It is a ubiquitous virus that infects a large percentage of humans worldwide, often at an early age. CMV spreads through a variety of ways, and virus is persistently expressed in epithelial cells resulting in virus excretion in bodily fluids, with infected saliva as its preferential vehicle. Other vehicles include breast milk, urine, genital secretions, and other body fluids, to a lesser extent.

As all members of this family, CMV establishes latent infection in specific body districts after primary infection, specifically in monocytes and bone marrow CD34^+^ myeloid progenitor cells. Viral reactivation occurs from time to time, under the influence of numerous factors. Latency is regulated by a variety of specific genes in the virus genome. However, CMV is thought to persist also due to its many ways of evading the host immune defenses [4, 5]. Several viral proteins, such as interleukin 10 homolog and others, create an immunosuppressive environment around infected cells that avoids elimination of the latently infected cell by the immune system [6]. Virus expression is kept under the control of the immune system. CD4^+^ T cells are considered key elements, as proven by the enormous reduction in the rate of CMV-related disease in HIV patients after the introduction of the highly active antiretroviral therapy (HAART) [7].

In the immunocompetent host, primary infection is almost always subclinical. However, symptoms resembling infectious mononucleosis by EBV can occasionally be present. Typically, most of the population gets infected during childhood and for this reason children in childcare are a population at risk of infection by exposure to saliva-contaminated objects. Primary infection is followed by viral excretion in urine and other body fluids for over 6 months [8]. Viremia drops when CMV-specific CD4^+^ T cells peak [9].

Vertical transmission of infection from a mother with active infection to the fetus is a cause of concern. Between 0.2% and 2% of newborns are infected with CMV in utero or perinatally and, of these, approximately 10% develop clinically evident disease [1, 10]. Approximately 90% of symptomatic newborns and 15% of the asymptomatic ones experience long term sequelae [11]; nowadays, CMV is the most common cause of hearing and neurological impairment due to nongenetic causes [10].

The risk of infection for a fetus of an actively infected mother has long been considered to depend on whether the mother has primary infection or reactivation [12]. Pregnant women with primary infection have higher chances of transmission during pregnancy, with a frequency of transmission of roughly a third [11]. Because in developed countries seroprevalence for CMV ranges between 40% and 60%, many women are at risk of acquiring primary CMV infection during pregnancy. The presence of antibodies is considered a factor that decreases both the risk of transmission of CMV during reactivation to the fetus and the chances of serious long term consequences of infection. Indeed, the risk of transmission drops to 1.4% as a consequence of reactivation during pregnancy [13]. Nevertheless, in developing countries, where almost everybody is seropositive for CMV, prevalence of congenital CMV infection ranges between 1% and 5% of births. Therefore, recently, reactivation of maternal CMV infection was recognized as being responsible for the majority of congenital CMV infections [14].

Contrary to the immunocompetent host, CMV infection often has serious consequences in the immunocompromized host. For solid organ transplantation (SOT) recipients, the risk of serious disease varies according to whether recipients are seropositive or not: patients who are seronegative before transplantation have a 40 to 80% risk of acquiring primary CMV infection from the graft [15, 16]. In these patients, CMV infection increases the risk of superinfection by many pathogens, and, most importantly, they are likelier to undergo graft rejection. On the other hand, patients who are seropositive before transplant are at a lesser risk of infection by the graft, but CMV reactivation may still have serious consequences when they are under immunosuppressive drugs. For these reasons, transplanted patients routinely undergo preemptive antiviral prophylaxis for at least 3 months after transplant, although a period of 6 months is recommended [17].

HIV patients, another category of immunocompromized patients, may experience retinitis, gastrointestinal and central nervous system end organ disease, and pneumonia due to CMV reactivation [18, 19]. The frequency of such events has been greatly reduced by HAART therapy.

## 3. Immune Response to CMV

A very large number of viral proteins are encoded by the relatively complex CMV genome. A total of 751 translated ORFs were recently identified by several experimental approaches, including next generation sequencing and high-resolution proteomics [20].

CMV infection activates robust responses to many of these proteins by the adaptative and innate immune system, but it has been somewhat hard to define proper correlates of immune protection.

Upon primary CMV infection, the earliest antibody response is directed against tegument protein [21]. Neutralizing antibodies are mounted after at least 2 months from infection and are directed against a number of envelope proteins, such as the gH/gL/UL128-131A complex and gB, gH, and gM/gN [22].

Antibodies have a relevant protective role during infection, in terms of both disease transmission and severity. Their presence in maternal blood has long been considered to lower the chances of fetal infection [12]. However, it is not clear to what extent antibodies are protective since their presence in maternal milk does not prevent transmission of infection to babies [23, 24]. In addition, a recent clinical trial aimed at studying fetal transmission of CMV infection by pregnant women with primary infection showed that infusion of hyperimmune globulin did not seem to prevent transmission to fetus [25].

One can conclude that antibody titer is a correlate of protection, but the causative link remains elusive. The fact that some women with high neutralizing Ab titers still transmit virus to their fetus and that pregnant women can be reinfected despite vigorous neutralizing antibody titers indicates that these responses do not absolutely prevent infection, although they undoubtedly reduce the potential for infection [12]. Recently, it has been shown that kinetics of antibody responses to different CMV targets are markedly different [26, 27]. These results make it tempting to speculate that the interplay between viral replication and the development of antibodies to very specific targets might be more relevant compared to the entire anti-CMV antibody titer.

Antibody titers may reflect the entity of CD4^+^ T cell responses, since they depend on T cell help in a stringent way. Indeed, very recent data on* Macacus rhesus* demonstrate that protection of the fetus from vertical transmission of CMV infection depends on CD4^+^ T cells [28]. Whether this depends on CD4^+^ T cells directly or indirectly is still to be clarified. In* Macacus*, a lower titer of neutralizing antibodies, in spite of a normal antibody titer, was observed in the absence of adequate T cell help [28].

It has been known for more than 20 years that helper and killer T cells are also expanded at extraordinarily high frequencies during CMV infection. Although CD4^+^ T cells seem to react preferentially against structural viral proteins and CD8^+^ T cells react mostly against immediate early ones, many proteins are recognized by both [29, 30]. Certain viral proteins are immunodominant, especially pp65 and IE-1; however, not all antigens seem to induce protective immunity. For example, it was shown that developing high frequencies of CD8^+^ T cells against IE-1, but not against pp65, early after transplantation is associated with protection from CMV disease [31]. These data are quite controversial, as they were not confirmed by several subsequent studies [32].

CMV-specific CD8^+^ T cells have long been known to be key in controlling viral replication in infected hosts and their adoptive transfer has proven therapeutical in transplanted patients [33, 34]. Though underestimated in the past, such T cell-mediated response seems to be quite relevant in the infected fetus as well [23]. A very recent study performed on pregnant women with primary CMV infection who either transmitted or did not transmit CMV to their fetus showed that proliferative ability of both CD4^+^ and CD8^+^ T cells and IL-2 secretion by CMV-specific CD4^+^ T cells are lower in women who transmit CMV [35].

Repeated reactivation of CMV progressively enhances the number of CMV-specific CD8^+^ T cells, which can accumulate in time up to 20% of total CD8^+^ T cells, in what is known as “memory inflation” [36]. CD4^+^ T cells are also expanded with similar kinetics, though to a lesser extent [37]. Interestingly, proteins expressed during latency are also recognized by T lymphocytes but these seem to quench immune reactivity rather than having an effector role [29].

*γδ* ^*+*^ T cells are also expanded during CMV infection in the adult and in the fetus [38], where expansion of V*δ*2^−^ *γδ* ^+^ T cells was shown to be a specific blood signature of CMV infection [39]. Notably, *γδ* ^+^ cells were proven to have a protective activity in SOT patients and in early life [23, 38].

Natural killer cells have long been known to be critical in recovery from CMV infection, both in adult and in fetal life [23]. As part of innate immunity, they have been shown to rapidly increase in primary infection until roughly 2 months after infection and then to decrease [35]. They have recently been demonstrated to be more similar to the adaptive arm of the immune system than first thought. Indeed, they seem to undergo some selection similarly to T cell development in the thymus; in addition, although they do not undergo somatic rearrangement of their receptor genes, they have been shown to expand in an antigen-specific fashion and establish a pool of “memory” cells in the mouse [40].

In humans, Rölle et al. showed that expansion of specific NK cell subsets during CMV infection occurs in an MHC-dependent way and relying on CD94 and NKG2C on NK cells and HLA-E and IL-12 derived by monocytes, although the antigen, if there is a specific one, is still unknown [41]. In turn, CMV devotes at least 6 ORFs to counteracting NK activity, although this is not their only function [42]. This underlines how important NK cells are in recovering from CMV infection.

To sum up, both humoral and cellular immune responses contribute to protection from CMV infection reactivation and to recovery. However, the specific role of each of the two arms of the immune response has not been defined, nor have the interactions of the two components. This consideration paves the way to future studies where antibody-dependent cellular cytotoxicity will have to be deeply investigated.

## 4. Most Commonly Used Methods Available for CMV Diagnosis: A Brief Overview

Because the consequences of CMV infection vary in different types of patients and treatment is readily available, it is important to diagnose active CMV infection. In many instances, the most important diagnostic questions are whether CMV viremia is due to primary infection or reactivation and what the chances of serious disease are. CMV infection can be diagnosed with practically all available methods in laboratory diagnostics, but different clinical questions can be solved with careful choice.

In the case of primary infection in adults, the search of serum antibodies is first choice to determine whether adults with infectious mononucleosis symptoms or pregnant women who have been exposed to CMV have been recently infected by CMV. CMV-specific antibodies can be determined by enzyme-linked immunosorbent assay (ELISA) in various formats as a first choice. ELISA can also be used to search for IgM [3].

The presence of CMV-specific IgM with low-titer and no IgG characterizes primary infection. Because IgM tends to be present also during reactivation, IgG is often found together with IgM; IgG-avidity tests may therefore be useful tools to differentiate primary infection from reactivation. As for other pathogens, avidity of IgG binding CMV increases with time from infection. When low, this index identifies recent CMV infection as opposed to reactivation [43].

In pregnant women, it is important to determine the risk of transmitting CMV infection to the fetus in utero, besides the occurrence of infection itself. Studies have shown that, surprisingly, cellular immunity is directly correlated to the risk of transmission [44, 45].

Direct determination of the presence of CMV virions is first choice in the diagnosis of CMV infection in the newborn and fetus, where maternal antibodies may render serology cumbersome. While intrauterine infection can be diagnosed by sampling amniotic fluid, newborns shed CMV in most body fluids, such as urine and saliva for months [46]. Isolation of CMV may be attempted by classical or shell-vial cell culture methods. This method has several drawbacks, above all the fact that CMV grows slowly and some clinical isolates do not easily adapt to culture conditions, thus leading to false negative results.

Molecular methods have largely replaced cell culture in many laboratories. Most of these are based on nucleic acid amplification of CMV DNA from blood, urine, bronchoalveolar lavage fluid, and other body fluids. Modern quantitative molecular methods mostly rely on real-time PCR for clinical viral load testing. Recently, digital droplet PCR (ddPCR) was evaluated as a method allowing precise direct quantification without requiring a calibration curve [47, 48]. It relies on limiting partition of the PCR volume in a large number of droplets, each of which may be envisioned as a PCR reaction giving a positive or negative result according to whether a single target molecule was present in the droplet or not. When compared to quantitative real-time PCR, ddPCR proved to reduce quantitative variability but was not as sensitive as real-time PCR [48].

These methods are more expensive than cell culture but are much faster and more sensitive. In addition, they may be rendered quantitative and are relatively independent of sample deterioration. Determination of T cell immunity may be helpful diagnosing infection in children younger than 12 months [49, 50].

Finding CMV-specific IgM in fetal and newborn serum may also be used to diagnose infection.

Consensus guidelines have been set for the management of SOT patients [17]. Before transplantation, CMV IgG should be determined in patients and donors because CMV^−^ recipients transplanted with tissues from CMV^+^ donors are at high risk of primary CMV infection. To discriminate equivocal serology results, it may be useful to assess cellular immune status against CMV. After transplantation, active disease, whether primary or due to reactivation, should be closely monitored by determining viral load. Classically, this has been carried out by the pp65 antigenemia test: purified peripheral blood leukocytes (PBLs) from patients are enumerated at UV microscope after staining with anti-CMV nuclear protein pp65 fluorescent monoclonal antibodies. A semiquantitative result of the number of PBLs with infected nuclei out of 200,000 is obtained. However, neither interlaboratory standard is available nor has common agreement on cutoff values been reached for this test. Most laboratories have replaced determination of antigenemia with quantitative determination of viral DNA load in whole blood or plasma.

Although a plasma DNAemia cutoff value of 10,000 copies/mL has been suggested before CMV therapy is started [51], a consensus has not been reached and cutoffs differ between laboratories. A WHO standard to calibrate diagnostic tests is now available [17]. Serology is not helpful in posttransplant patients, where negative results may be due to immune suppression.

## 5. Diagnostic Methods Based on T Cell Reactivity

While the presence of CMV-specific IgG has been long considered the golden standard to define infection by CMV, it has been proposed that measuring CMV-specific T cell responses by specific assays might help predict whether a patient will develop serious CMV disease after transplant. Improving criteria to treat patients for active CMV infection would avoid unnecessary treatment and related toxicity and allow saving the costs of repeated monitoring of CMV reactivation and of prophylaxis. Although at present it is not routinely performed, monitoring cell immunity to CMV, alone or in parallel to viral load determination, may help identify patients that actually require treatment for CMV disease after transplantation and/or help set personalized cutoff values for CMV DNAemia or antigenemia for patients at high or low risk of CMV disease, that is, with low or high T cell responses to CMV, respectively.

CMV-specific T cell reactivity before transplantation, and not so much seropositivity, was shown to inversely correlate to the risk of CMV viremia and disease after transplant; spontaneous clearance of CMV may occur in patients with positive CMV DNAemia or antigenemia, especially in patients where robust T cell immunity can be detected against CMV, independently of their serological status against CMV [52].

In the past years, different techniques have been developed to detect antigen-specific T cell response: flow cytometry-based multimer or intracellular cytokine staining (ICS) and ELISpot have been employed in research and in clinical studies [53, 54]. The advantages and disadvantages of each technique have been highlighted in a comparative study [55].

ICS for CMV-specific T cells followed by flow cytometry is particularly useful in basic research [54]. This assay consists of a 4–6 h stimulation of PBL or whole blood with CMV antigen, costimulatory antibodies, and a secretion inhibitor like Brefeldin A. This is followed by fixation, permeabilization, and staining with antibodies against an intracellularly retained cytokine, most commonly IFN*γ*, and surface markers like CD4, CD8, and others of interest, labeled with different fluorophores. FACS analysis is then performed. The assay allows enumeration of CMV-specific T cell subsets and simultaneously permits determination of the phenotype of single cells and the cytokines they produced after stimulation. ICS is a very informative test because it allows the detection of both CD8^+^ and CD4^+^ antigen-specific T cells in a single assay. A broad spectrum of different phenotypic markers and cytokines can also be analyzed at the same time [54]. It may be useful to detect immune responses also in samples of patients who lack response against particular dominant epitopes in their pathogen-specific T cell repertoire [56]. However, ICS requires a good level of expertise and is labour intensive, in addition to being costly.

A variation of the ICS can be carried out by the use of tetramers of MHC class I and CMV-derived peptide, used in ICS both as stimulants of specific CD8^+^ T cells and as fluorescent labels [53]. Recently, this technique was used in an effort to define cutoff values of CMV-specific CD8^+^ T cell in allogeneic hematopoietic stem cell transplant recipients associated with protection from recurrent or persistent CMV infection. Recovery from CMV disease was shown to be faster when these cells were ≥ 7 cells/*μ*L of blood during the first 65 days after transplantation, whereas a value < 7 cells/*μ*L was indicative of CMV-related complications [57].

More recently, the tetramers technique have been improved by using “multimers” which have higher affinity for the T cell receptor (TCR) compared to tetramers and guarantee more stable binding. Multimer staining allows labeling, visualization, and enumeration of peptide-specific T cells in patient peripheral blood samples. Even if this test requires a small amount of blood sample and the results are available within 3 hours, it is not suitable as a diagnostic tool, because several multimers are required to obtain a full overview of the immune response for each patient. Furthermore, due to MHC polymorphism, a multimer for each single HLA allele would be necessary [58].

Because of their complexity, both methods are limited to research activities.

An ELISPOT-based method to determine cellular immune reactivity against CMV was developed by Oxford Immunotec. It uses a mixture of peptides derived from CMV antigens IE-1 and pp65 that stimulate IFN*γ* secretion by both CD4^+^ and CD8^+^ T cells from purified PBL. Individual IFN*γ*-producing cells are enumerated out of a total of 200,000 PBLs placed in each ELISPOT well coated with antibodies capturing secreted IFN*γ*. Controls include unstimulated (negative) and phytohemagglutinin- (PHA-) stimulated (positive) cells.

ELISPOT allows discriminating between low responders (20–50 spots/10^6^ PBLs) and high responders (>100 spots/10^6^ PBLs) in 2 days from blood sampling [59]. This assay was shown to be useful to predict the risk of CMV reactivation and infection in hematopoietic and kidney transplant recipients, since strong cell immunity is predictive of less serious CMV disease [60, 61]. This assay was also used to show that higher values of cell-mediated immunity in the blood of pregnant women are associated with risk of fetal CMV transmission [44, 62]. This surprising observation might be due to the fact that the number of specific T cells increases with viremia. However, experimental data do not agree with these findings and demonstrate that the number of CD4^+^ T cells correlates with protection [28].

The assay linearity has been shown to be comparable to ICS [55] but ELISPOT for CMV-specific T cells was shown to be more sensitive than ICS [63].

ELISpot is, so far, the most sensitive method to determine T cell frequencies, but it is costly and laborious and, above all, it is hard to standardize for clinical purposes because ranges of frequencies vary greatly in the general population.

QuantiFERON-CMV (QT, Qiagen) is an assay that measures IFN*γ* release after stimulation of CD8^+^ T cells in whole blood with a cocktail of peptides binding a range of different HLA-I haplotypes, designed on the basis of a variety of CMV proteins. Again, controls are stimulation with phytohemagglutinin or no stimuli [64]. One mL of heparinized whole blood is incubated for 15–24 h, and then IFN*γ* content is measured in plasma by ELISA. It is CE marked (i.e., approved by the European Community and legally placed on the market in Europe) for clinical diagnostic use in Europe but not yet FDA approved in the United States. The assay yields positive and negative results when >0.2 or <0.2 International Units (IU) of IFN*γ*/mL, respectively, is recovered from CMV-stimulated supernatants. The result is obtained after subtraction of the value of the unstimulated sample and only if the value in PHA-stimulated blood is >0.5 U/mL. In addition, an indeterminate result can be obtained when no IFN*γ* can be found in the CMV or in the PHA-stimulated blood, where patients have low numbers of PBLs. The advantage of QT is its ease in clinical application, requiring minimal sample processing and technical expertise. However, the determination of IFN*γ* release by CD8^+^ T cells alone may be a limitation, since CD4^+^ T cells are as relevant. In addition, the use of peptides may lead to false negative results for rare HLA haplotypes.

Positive QT results in the week of the onset of CMV reactivation or, alternatively, 2 months after allogeneic hematopoietic stem cell or kidney transplantation were shown to correlate with a lower risk of complicated reactivation [60, 65]. Among QT-positive patients, roughly 6% develop CMV disease, versus 22% of QT-negative ones. The group with the highest risk of CMV reactivation was the indeterminate group, where 58% of patients developed CMV disease at 12 months after transplantation [66].

Although QT was shown to be as good as ELISPOT in determining the risk of CMV infection in kidney transplant recipients [60], results from QT analysis were not found to correlate to the risk of transmission of congenital CMV, as opposed to ELISPOT [44].

Transplant patients with positive results have been shown to have a reduced risk of CMV reactivation or of severe disease upon discontinuation of antiviral prophylaxis [65, 67].

QT-CMV was compared to ELISPOT performed with a CMV pp65 peptide mix (10 *μ*g/mL; AID) in monitoring CMV cell immunity in kidney transplant patients. The assays were shown to display good correlation and similar sensitivities and specificities [60]. However, both assays in their present format failed to detect all CMV-reactive individuals and would profit from improvement of the selected antigen. Whole CMV virions were proposed to overcome failure of certain HLA types to bind the mixture of pp65 peptides [59]. In contrast, another study compared the usefulness of ELISPOT and that of CMV-QT in predicting transmission of congenital CMV infection by pregnant women. In this study, mother CMV-specific T cell frequencies in mothers determined by ELISPOT, but not by QT, correlated with congenital CMV, together with maternal viremia and viruria (*p* < 0.05) and correlated negatively with CMV IgG-avidity (*p* < 0.01) [44].

In comparison to ICS, QT is as specific but less sensitive [68]. However, ICS is definitely more difficult to standardize and not suitable for automation.

## 6. Concluding Remarks

CMV infection may represent a risk for transplanted, immunocompromized, or immunologically immature individuals. In transplanted patients, measuring viremia levels for CMV is useful to determine whether the virus has reactivated due to immune suppression. However, patients may or may not clear CMV spontaneously, and therefore more detailed clinical diagnostic tools are warranted to set personalized cutoffs for antiviral therapy, given that laboratory assays, such as the determination of antibody titer, isotype, and avidity, are of limited utility to assess risk of transmission of infection from mother to fetus. For these reasons, many clinical studies have attempted to measure CMV-specific T cell reactivity as an indicator to predict the outcome of infection in different clinical situations. Indeed, most studies agree that quantifying T cell response to CMV can be useful and at least one assay, QT, has been standardized for clinical diagnostic use. Future milestones may be as follows: (1) first future milestone is to extend observations concerning the benefits of determining T cell reactivity in different clinical situations; (2) second one is to improve the currently available methods, for example, by allowing QT to determine CD4^+^ T cell reactivity besides CD8. This may be achieved by including whole recombinant CMV antigens, virions, or defined MHC class II-binding peptides as stimulators of IFN*γ* release and may lead to more reliable and generalized cutoffs for antiviral therapy. (3) The third future milestone is to adapt methods, that is, CMV ELISPOT, which may be more sensitive than others, to the clinical practice.
